# Supplementary material for: Chromosome-Wide Distribution and Characterization of H3K36me3 and H3K27Ac in the Marine Model Diatom Phaeodactylum tricornutum
Source: Plants (Basel). 2023 Aug 2;12(15):2852. doi: 10.3390/plants12152852 (PMC10421102; doi:10.3390/plants12152852)
Supplement: Supplementary file 1 [file plants-12-02852-s001.zip › plants-2476864-supplementary.pdf]

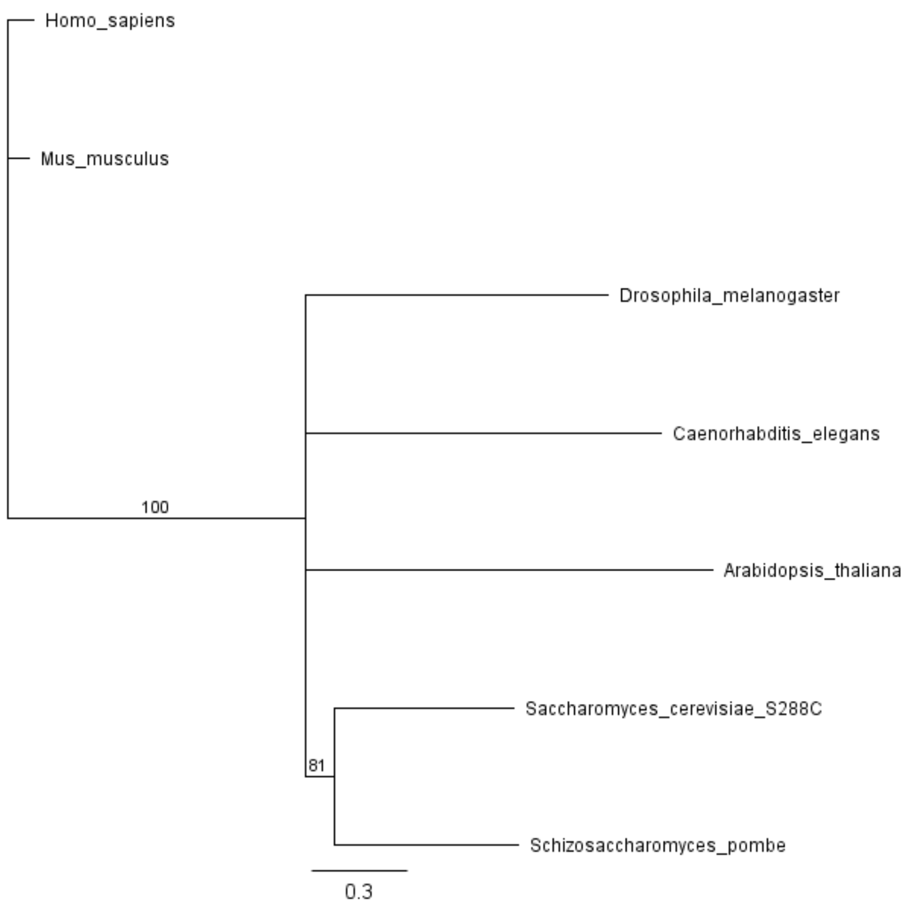

Supplementary Figure S1

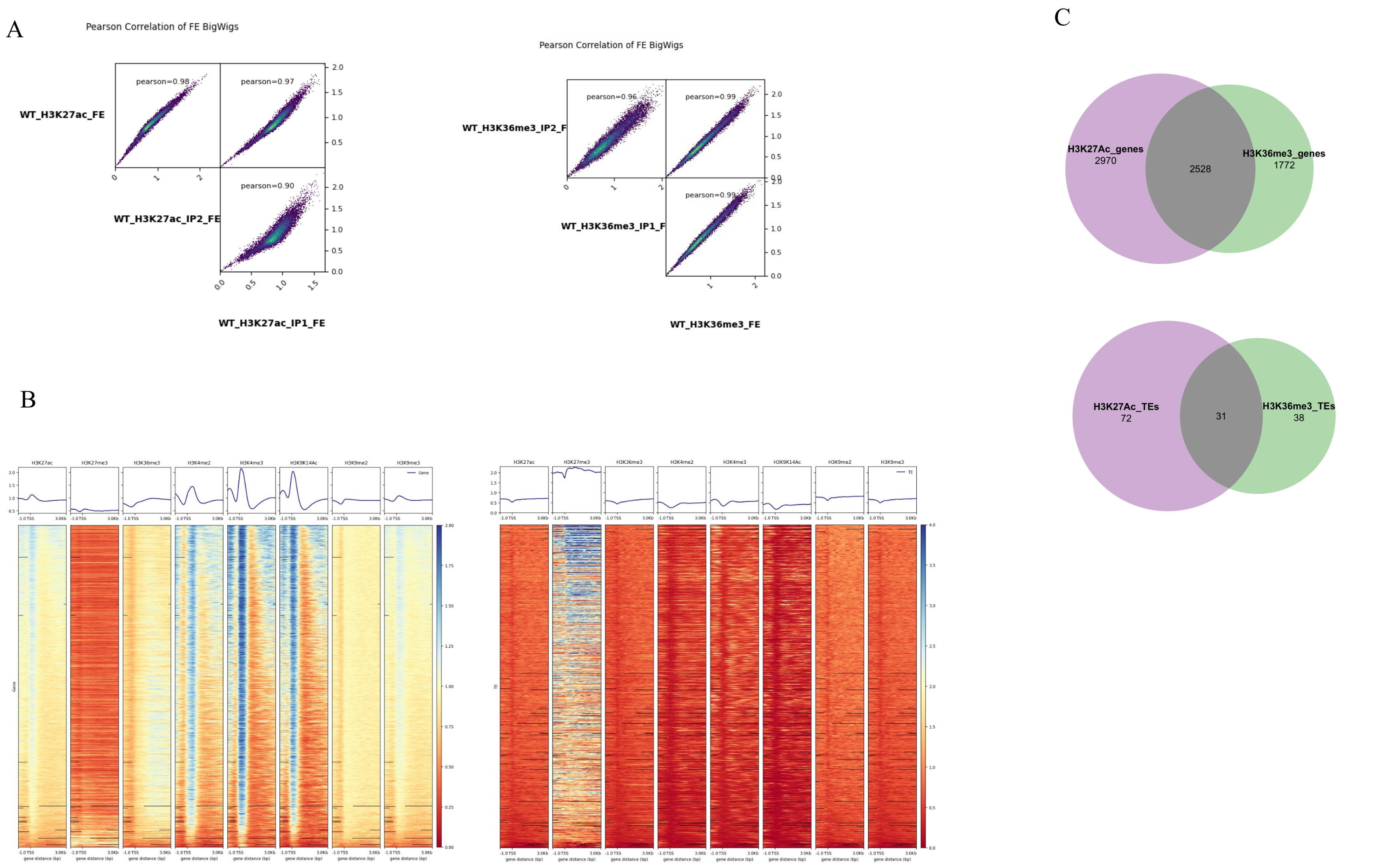

Supplementary Figure S2

	Genome occupancy		Detected Peaks		Peaks Annotation	
	Genome coverage (Mb)	Genome coverage (%)	Number	Mean Size (bp)	Genes	TEs
**H3K27ac**	3.68	13.24	4928	747	5498	103
**H3K36me3**	6.41	23.05	3400	1885	4300	69

Supplementary Table S1

Primer names	Sequence
J43430 Fwd	AGTTCGAGACGTCCATTGGT
J43430 Rev	TGGTGCCATTGTAGTAGCCT
J45714 Fwd	CTCGATAGGAACGGACCA
J45714 Rev	CGTCTGTGCTGATGGTATGG
J50371 Fwd	GGGACACGACTCGAAACG
J50371 Rev	CCGTTGTTCTTGTCGTGGTGG
EG01064 Fwd	GCTATCACTACATTGGCCCAC
EG01064 Rev	CGCTCACATCCATCCACGTT
Q-Act12 Fwd	TCGCCTGAGTCGAGAACACA
Q-Act12 Rev	GCCCATCCAGTCCTGTTGAC
Q-rps Fwd	CGAAGTCAACCAGGAAACCAA
Q-rps Rev	GTGCAAGAGACCGGACATACC
Q-TubA Fwd	CTGGGAGCTTTACTGCTTGGA
Q-TubA Rev	ATGGCTCGAGATCGACGTAAA

Supplementary Table S2
